# Compressive Dorsal Myelopathy Secondary to Extramedullary Hematopoiesis in a Thalassemic Patient

**DOI:** 10.1155/2019/5827626

**Published:** 2019-10-17

**Authors:** Ismail Ibrahim Ismail, Fathi Massoud, K. J. Alexander, Jasem Youssef Al-Hashel

**Affiliations:** ^1^Department of Neurology, Ibn Sina Hospital, Kuwait; ^2^Department of Medicine, Faculty of Medicine, Health Sciences Centre, Kuwait University, Kuwait

## Abstract

**Background:**

Extramedullary hematopoiesis (EMH) is one of the rare causes of spinal cord compression (SCC). It results from noncancerous proliferation of hematopoietic tissue outside the bone marrow as a compensatory mechanism for ineffective erythropoiesis. It occurs in the paraspinal area in 11–15% of thalassemic patients in intermediate and severe cases causing a paraspinal compressive mass. We present a rare case of spinal EMH with thoracic cord compression in a 22-year-old female with beta thalassemia who presented with paraparesis and we provide a review of literature.

**Case Report:**

A 22-year-old female patient with a known history of beta thalassemia presented with subacute onset of weakness and numbness of both lower limbs with a sensory level at T6. Magnetic resonance imaging (MRI) of the dorsal spine showed cord compression secondary to paraspinal EMH from T2 to T9 with most prominent compression over T5. She was managed with blood transfusion and low-dose radiotherapy.

**Conclusion:**

Although rare, EMH should be suspected in thalassemic patients presenting with paraplegia. Treatment with blood transfusions is usually effective. Other options include radiotherapy, surgery, hydroxyurea or a combination of these modalities.

## 1. Introduction

Thalassemia is an autosomal recessive blood disease which leads to a chronic anemic state. It is classified into *α*-thalassemia and *β*-thalassemia according to which chain of the hemoglobin molecule is affected. Non-transfusion-dependent thalassemia (NTDT) is a state that does not require regular blood transfusion, contrary to transfusion-dependent thalassemia (TDT) that is usually treated with repeated blood transfusions and iron chelation. The clinical phenotype and severity of *β*-thalassemia can manifest as major, minor, or intermedia. Bone marrow transplantation is curative and despite being expensive, it is usually offered to thalassemic patients in Kuwait [1].

EMH is a well-documented complication of conditions associated with ineffective hematopoiesis resulting in a chronic anemic state. The noncancerous proliferation of hematopoietic tissue outside the bone marrow causes expansion into nearby normal tissues. Paraspinal EMH affects 11–15% of thalassemic patients and is usually asymptomatic in the majority of them [2].

We report a rare case of spinal EMH with dorsal cord compression and myelopathy in a 22-year-old female with beta thalassemia who presented with paraparesis and we provide a review of literature.

## 2. Case Report

A 22-year-old Kuwaiti female with a past history of non-transfusion dependent beta-thalassemia who do not receive any current active treatment presented with a two months history of subacute onset of gait difficulty secondary to weakness and stiffness of both lower limbs with recurrent falls. Her condition was associated with back pain, mainly in the thoracic region and bilateral numbness of the lower part of her body up to the level of the costal margin. She had no sphincteric affection, no history of trauma or fever and no history of previous similar condition.

Neurological examination showed a fully conscious and oriented patient with normal speech, higher mental functions and cranial nerves. Motor examination was normal in both upper limbs. Lower limbs examination showed spastic paraparesis with hypertonia, power of grade 4+/5, hyperreflexia (3+) and bilateral extensor plantar response. Sensory examination showed a sensory level at T6 with poor joint position and movement sensation. She had no cerebellar signs while her gait was spastic.

Laboratory investigations were as follows: hemoglobin (Hb): 66 g/L, Hematocrit (Hct): 0.198, mean corpuscular volume (MCV): 63.2 and mean corpuscular hemoglobin (MCH): 22.2. Iron level was 25.7 umol/L, transferrin: 1.4 g/L, ferritin: 39 ng/mL and saturation: 74%. Serum electrolytes, renal function, liver function, coagulation profile, vasculitic work-up and thyroid function were normal.

**Figure 1:**
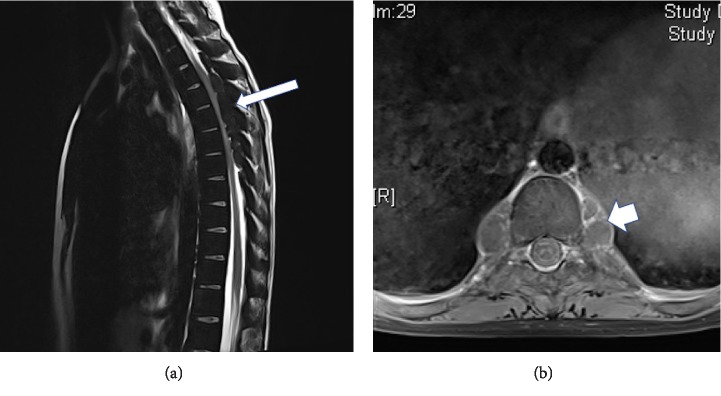
MRI of the dorsal spine: (a) sagittal T2-weighted, (b) axial T1-weighted images with contrast showing posterior epidural multilobulated extramedullary hematopoietic mass from T2 to T9 (arrows) with the largest at T5 causing the most prominent cord compression and showing no contrast enhancement.

MRI of the brain was normal. MRI of the spine showed multi-lobulated soft tissue masses at the posterior epidural spaces of the thoracic spine from T2 to T9, with the most prominent cord compression at T5 level. These masses are isointense to bone marrow and show no significant enhancement suggestive of extramedullary hematopoiesis (Figure 1).

**Figure 2:**
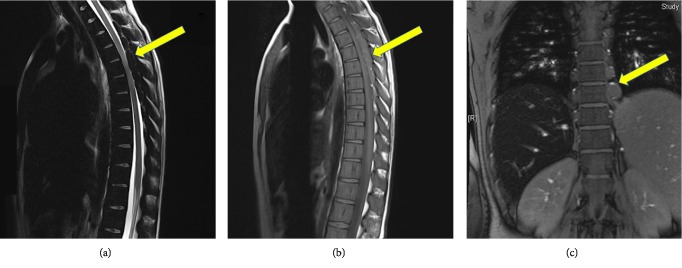
Follow up MRI of the spine after blood transfusion: (a) sagittal T2-weighted, (b) sagittal T1-weighted, (c) coronal T1-weighted images showing interval reduction in cord compression.

She was evaluated by hematologist and she received blood transfusion in the form of one unit of packed red blood cells. Hemoglobin level increased from 6.6 to 9.1 g/L. She showed partial clinical improvement after transfusion. Follow up MRI the following day showed reduction in cord compression (Figure 2).

She was further evaluated by neurosurgery who refrained from surgical management due to initial improvement and Oncologist advised for adjuvant low dose radiotherapy to prevent recurrence. She was also put on scheduled blood transfusions and she remained clinically and radiologically stable on follow up visits for one year with improved strength in both lower limbs (G 5/5), 2+ reflexes and down-going plantar response bilaterally.

## 3. Discussion

EMH has been observed in numerous hematological disorders, including thalassemia, sickle cell anemia, hereditary spherocytosis and idiopathic thrombocytopenic purpura. It occurs as a response to chronic anemia states as a homeostatic mechanism via the production of erythropoietin [3].

The carrier rates of *β*-thalassemia in the Arab countries range from 1% to 11% of the population [4]. In Kuwait, it is estimated that the number of thalassemic patients are more than 475 cases [5, 6]. Galanello et al. estimated the percentage of *β*-thalassemia carriers in Kuwait to be 3% of population [7].

The association between EMH and NTDT is well-recognized and Gatto et al. was the first to report SCC in these patients in 1954 [8]. The incidence of EMH in thalassemia vary in literature between 0.8% in thalassemia major and up to 20% in thalassemia intermedia. EMH is particularly common in NTDT patients (20%) and rare in TDT (<1%) due to underlying bone marrow suppression from repeated transfusions [9, 10].

It affects the paraspinal region in 11–15% of the cases followed by the liver, spleen, and lymph nodes [11]. The most frequent location of paraspinal EMH is thoracic and it is usually asymptomatic and incidentally discovered on imaging in the majority of cases with only 20% presenting with SCC and significant disability [12]. The reason why there is a predilection for thoracic cord region is unclear, however, some authors believe that stem cells could have leaked into the thoracic epidural space from the expanding bone marrow [13].

Paraspinal EMH is more common in males with a M:F ratio ranging from 2.5 : 1 to 5 : 1 and occurs mainly in chronic cases in the third and fourth decades of life. The clinical manifestations depend on the site and size of the mass and include: back and lower limb pain, paresthesia, paraplegia, paraparesis and sphincteric dysfunction [14].

The gold standard for diagnosis is MRI as it can show clearly the site and extent of EMH and can differentiate between active and inactive lesions. Active lesions contain blood while inactive lesions contain fat and iron deposits. Active recent lesions show intermediate signal intensity in T1 and T2 weighted images while inactive lesions show high signal intensity in both T1 and T2 weighted images if it contains fatty tissue or low signal intensity if it contains iron deposition. Gadolinium enhancement varies in literature between no/minimal enhancement to marked enhancement [15]. In our patient, the lesion was isointense to bone marrow with no contrast enhancement.

Treatment of paraspinal EMH should be individualized, in the absence of large randomized controlled trials and evidence-based guidelines in literature. Management modalities include repeated blood transfusions with iron chelation, radiotherapy, surgical decompression, hydroxyurea, or a combination therapy depending on the size of the mass, severity of symptoms and clinical status [16, 17].

In patients presenting with acute cord syndrome, surgical interreference is warranted. An open approach with laminectomies and multi-level decompression is known to be effective. An alternative approach is minimally invasive incisions along the posterior midline with each incision being used to reach four adjacent levels [18].

In our patient, a conservative approach with a combination of blood transfusion and radiotherapy was performed. The mass regressed in size after one unit of blood transfusion due to elevation of hemoglobin level to 9.1 g/dl. This was followed by low-dose radiation (20 Gy in 10 fractions) as adjunctive therapy as hematopoietic tissue is extremely radiosensitive. In literature, up to 50% of patients shows neurological improvement within 3–7 days of radiotherapy.

## 4. Conclusion

Paraspinal EMH must be suspected in any thalassemic patient presenting with paraplegia. MRI is the modality of choice in diagnosis and evaluation of SCC secondary to EMH. Early diagnosis and management can prevent irreversible neurological damage.
